# Harnessing Novel Soil Bacteria for Beneficial Interactions with Soybean

**DOI:** 10.3390/microorganisms11020300

**Published:** 2023-01-23

**Authors:** Osiel Silva Gonçalves, Thamires Santos Souza, Guilherme de Castro Gonçalves, Alexia Suellen Fernandes, Tomás Gomes Reis Veloso, Sumaya Martins Tupy, Ediones Amaro Garcia, Mateus Ferreira Santana

**Affiliations:** 1 Institute for Global Food Security, School of Biological Sciences, Queen’s University Belfast, Belfast BT9 5DL, UK; 2 Grupo de Genômica Evolutiva Microbiana, Laboratório de Genética Molecular de Microrganismos, Departamento de Microbiologia, Instituto de Biotecnologia Aplicada à Agropecuária, Universidade Federal de Viçosa, Viçosa CEP 36570-900, MG, Brazil

**Keywords:** biocontrol, drought, genomics, soybean

## Abstract

It is claimed that one g of soil holds ten billion bacteria representing thousands of distinct species. These bacteria play key roles in the regulation of terrestrial carbon dynamics, nutrient cycles, and plant productivity. Despite the overwhelming diversity of bacteria, most bacterial species remain largely unknown. Here, we used an oligotrophic medium to isolate novel soil bacteria for positive interaction with soybean. Strictly 22 species of bacteria from the soybean rhizosphere were selected. These isolates encompass ten genera (*Kosakonia*, *Microbacterium*, *Mycobacterium*, *Methylobacterium*, *Monashia*, *Novosphingobium*, *Pandoraea*, *Anthrobacter*, *Stenotrophomonas*, and *Rhizobium*) and have potential as novel species. Furthermore, the novel bacterial species exhibited plant growth-promoting traits in vitro and enhanced soybean growth under drought stress in a greenhouse experiment. We also reported the draft genome sequences of *Kosakonia* sp. strain SOY2 and *Agrobacterium* sp. strain SOY23. Along with our analysis of 169 publicly available genomes for the genera reported here, we demonstrated that these bacteria have a repertoire of genes encoding plant growth-promoting proteins and secondary metabolite biosynthetic gene clusters that directly affect plant growth. Taken together, our findings allow the identification novel soil bacteria, paving the way for their application in crop production.

## 1. Introduction

Plants are intimately intertwined with microbial communities in which several distinct mechanisms mediate dynamic ecological interactions. Plants release photosynthates belowground through mucilage and exudates, which are used as energy sources by distinct dwelling microbial taxa Berg [1,2,3]. In return, some specific microbial taxa can promote plant growth and/or offer protection against biotic and abiotic stressors. They occur via the synthesis of phytohormones, acquisition of nutrients, and antagonistic interactions with plant pathogens.

It is estimated that less than 1% of bacterial species have been cultivated under laboratory conditions, a phenomenon known as the “Great Plate Count Anomaly” [4]. Soils are by far the richest environment, containing an extensive and diverse set of bacteria, of which the majority are yet unknown and are mainly detected by metagenome analysis [5,6]. Hence, the ecological features of most soil bacterial taxa, including their environmental preferences, phenotypes, and metabolic capacities are mostly unknown.

Apart from the rhizosphere, most of the soil is considered an oligotrophic environment. This area is distinguished by lower levels of microbial density and activity than those in high-resource environments [7]. The microbiome that inhabits this habitat is classified as a k-strategist, which implies that it can survive in low-nutrient conditions, grow at a slower rate, and has a high tolerance to toxic compounds [8,9]. Yet, most of the cultivation methods used in microbiology rely on nutrient-rich media, which may limit the study of oligotrophic bacteria from soil ecosystems [10]. These bacteria play key roles in regulating terrestrial carbon dynamics, nutrient cycles, and plant productivity. Therefore, novel strategies for assessing this unknown biological diversity are necessary.

Studies have shown the potential for cultivation of previously ‘unculturable’ bacteria from environmental samples using simple cultivation strategies [11,12]. The cultivation of ‘unculturable’ bacteria can be improved by combining oligotrophic media, extended incubation periods, and selection of slow-growing bacteria [12,13]. The goal of this study was to isolate and identify novel microbial species with the potential to plant growth-promoting rhizobacteria (PGPR) for soybean plants, as well as to apply genomics approaches to gain insights into bacteria–plant interactions.

## 2. Materials and Methods

### 2.1. Soil Sampling, Preparation of Media, and Isolation Procedures

**Figure 1:**
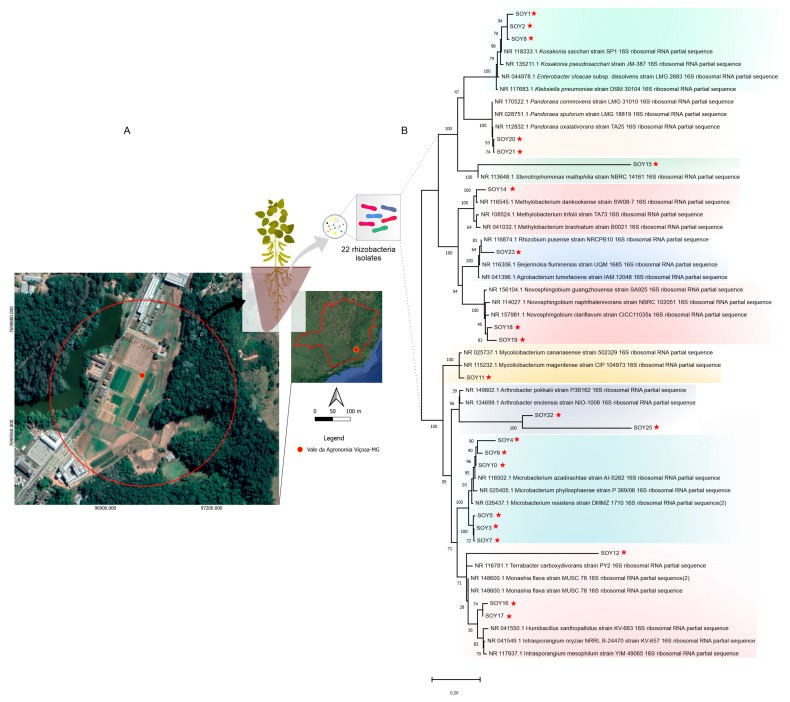
Study site of sample collection and phylogenetic tree of 22 rhizobacteria isolated from soybean. (**A**) The experimental field at the Universidade Federal de Viçosa (UFV), Minas Gerais, Brazil (20°46′01.1″ S 42°52′10.2″ W), indicated by the sampling sites in the red dot. The map was built using QGIS 3.16 Hannover (DATUM: SIRGAS2000, UTM Zone 22S). (**B**) Likelihood phylogenetic tree of 22 rhizobacteria. The evolutionary history was inferred using the maximum likelihood tree (1000 bootstrap replicates) and the general time-reversible substitution model + gamma distribution with invariable sites (G + I). The scale bar at the bottom indicates the number of differences in base composition among the sequences. Red stars indicate the 22 isolates described herein.

Five equidistant samples of rhizosphere soil from soybean (*Glycine max*) were obtained from the experimental field at the Universidade Federal de Viçosa, Minas Gerais, Brazil (20°46′01.1″ S 42°52′10.2″ W). The map was built using QGIS 3.16 Hannover (DATUM: SIRGAS2000, UTM zone 22S) (Figure 1A). Ten g of each sample was diluted in series with sterile saline. Aliquots of each dilution were inoculated in the following media: VL55 medium, 3.0 g of 2-[N-morpholino]ethanesulfonic acid (MES), 0.4 mM of MgSO_4_, 0.6 mM CaCl_2_, 0.4 mM of (NH_4_)_2_HPO_4_, 2 mL of tungstate/selenite solution (composition in 1 L of distilled water: 0.5 g of NaOH), 3 mg of Na_2_SeO_3_.5H_2_O, 4 mg of Na_2_WO_4_.2H_2_O [14] and 2 mL of SL-10 trace elements (composition in 1 L of distilled water: 10 mL of HCl (25%, vol/vol)), 1.5 g of FeCl_2_.4H_2_O, 70 mg of ZnCl_2_, 100 mg of MnCl_2_.4H_2_O, 6 mg of H_3_BO_3_, 190 mg of CoCl_2_.6H_2_O, 2 mg of CuCl_2_.2H_2_O, 24 mg of NiCl_2_.6H_2_O, and 36 mg of Na_2_MoO_4_.2H_2_O. The pH was adjusted to 5.5 with a mixture of 200 mM NaOH and 100 mM KOH. This base medium was autoclaved at 121 °C for 20 min and cooled to 56 °C. A 10 mL aliquot of 5% (*w*/*v*) xylan from beechwood (Fluka), 2 mL of vitamin solution (see below), and 2 mL of an SL-10 trace element solution [15] were added per 1 L. Selenite/tungstate, vitamin, and trace element solutions was sterilized by filtration. The vitamin solution contained (per 1 L of distilled water) 2 mg (+)-biotin, 2 mg of folic acid, 10 mg of pyridoxamine hydrochloride, 5 mg of thiamine chloride, 5 mg of riboflavin, 5 mg of nicotinic acid, 5 mg of hemicalcium D-((+)-pantothenate, 0.1 mg of cyanocobalamin, 5 mg of 4-aminobenzoate and 5 mg of DL-6,8-thiotic acid. Nystatin (10 mg L^−1^) was added. Plates with five replicates per sample were incubated in polyethylene bags to prevent desiccation at 25 °C in the dark for two weeks. The colonies were collected and transferred to fresh *DMSZ acidiphilium* medium agar (composition in 1 L of distilled water: 2 g (NH_4_)_2_SO_4_, 0.1 g KCl, 0.5 g K_2_HPO_4_, 0.5 g MgSO_4_ × 7H_2_O, 0.3 g yeast extract, 1 g D-glucose, 15 g agar–agar) plates for purification. The cells were grown in liquid DMSZ *acidiphilium* medium for cryostock preparation with stocks prepared with glycerol and maintained at −80 °C.

### 2.2. DNA Fingerprinting

For molecular characterization of bacterial isolates, molecular markers BOX, ERIC, and GTG. PCR was performed for fingerprint analysis. BOX GTG regions were amplified by PCR using primers BOXA1R (5′-CTACGGCAAGGCGACGCTGACG-3′) [16,17], and ERIC1R (5′-ATGTAAGCTCCTGGGGATTCAC-3′), ERIC2 (3′-AAGTAAGTGACTGGGG TGAGCG-5′) [17], and GTG5 (GTGGTGGTGGTGGTG). PCR reactions were adjusted to a volume of 25 μL containing 5.0 μL of Buffer, 2.5 μL of MgCl_2_, 2.5 μL of primer (BOX and GTG), 2.0 μL of genomic DNA 1.0 μL of dNTP, 0.25 μL of Taq DNA polymerase, and 11.75 μL of H_2_O. The regions were amplified with an initial denaturation step at 95 °C for 5 min, followed by 30 cycles at 94 °C for 1 min, 51 °C for 1 min, and 65 °C for 8 min, and a final extension at 65 °C for 8 min. The amplification products were analyzed by electrophoresis on 1.5% agarose gel, and the fingerprint patterns were analyzed using BioNumerics (Applied Maths) software, and the same pattern fingerprint was considered a clone.

### 2.3. DNA Extraction and 16S rRNA Sequencing, Processing, and Analysis

The isolates were inoculated into tubes with 20 mL of liquid DSMZ medium for one week at 28 °C and shaken on an orbital shaker at 180 rpm. Cells were collected by centrifugation and Genomic DNA was extracted using the Wizard^®^ Genomic DNA Purification Kit (Promega Corp., Madisonm WI, USA), as recommended by the manufacturer. DNA quality was checked using a NanoDrop 2000 (Thermo Fisher Scientific, Waltham, MA, USA) and subjected to gel electrophoresis (0.8% of agarose). The 16S rRNA genes were amplified using the 27F and 1492R [18]. PCR reactions were adjusted to a volume of 25 μL containing 0.1 μM of each 16SrRNA primer, 25 ng of genomic DNA, 0.2 mM of each dNTP, and 1.25 U of Taq DNA polymerase. The regions were amplified under the following conditions: initial denaturation at 95 °C for 5 min, followed by 34 cycles of 94 °C for 50 s, 60 °C for 1 min, 72 °C for 1 min 30 s, and a final extension at 72 °C for 8 min. Amplification products were analyzed by electrophoresis on a 1.5% agarose gel. The amplicon was sequenced in the ABI 3730xl System (Macrogen, Inc., Seoul, South korea).

The sequences were trimmed and assembled using Geneious Prime 2022.0.1 (Biomatters, Inc., Boston, USA). Next, the sequences were compared to the National Center for Biotechnology Information using BLASTn [19] against the 16S ribosomal RNA sequence (Bacteria and Archaea) database. We retrieved whole 16S rRNA sequences for each species’ family taxa and built an in-house database. These databases were aligned and subjected to phylogenetic tree inference using the neighbor-joining method in MAFFT version 7 (https://mafft.cbrc.jp/alignment/server/large.html [20]. The 16S rRNA sequences were then directly aligned against closely related strains in the Reference RNA sequence (Refseq) database retrieved for phylogenetic analysis. Next, the sequences were aligned using the ClustalW algorithm [21] in Mega11 [22]. The best-fit substitution model was calculated in Mega11, and phylogenetic trees were constructed using the maximum likelihood tree (1000 bootstrap replicates) and the substitution model general time revesible + gamma distribution with invariable sites (G + I).

### 2.4. Screening of Bacterial Isolates for In Vitro Plant Growth-Promotion Traits and Biocontrol

#### 2.4.1. Bacterial Growth under Reduced Water Availability

Isolates were grown in Tryptone Soya Agar (TSA) medium (10%) with additional sorbitol at five different concentrations (0 g/L^−1^; 85 g/L^−1^, 285 g/L^−1^, and 660 g/L^−1^) to simulate water stress at 25 °C [23].

#### 2.4.2. Exopolysaccharide Production

The isolates were inoculated onto 5 mm diameter paper discs disposed of in a DSMZ medium. The production of exopolysaccharide was checked by slime appearance and mixing a portion of the mucoid substance in 2 mL of absolute ethanol, in which the formation of a precipitate indicated the presence of EPS [24].

#### 2.4.3. Indole Acetic Acid (IAA) Production

Aliquots of 100 μL of bacteria were initially grown in 10 mL of TSA medium (10%) for 48 h in the dark at 28 °C. Next, colonies were transferred to fresh plaque-containing TSA medium (10%) supplemented with 5 mM L-tryptophan. The colonies were covered with a cellulose filter (0.45 μm pore size), and incubated in the dark at 25 °C. After 48 h, the membranes were washed with Salkowski reagent (50 mL of perchloric acid (35%) and 1 mL of FeCl_3_ solution (0.5 M) for 30 min in the dark [25]. The appearance of pink to red indicates IAA production.

#### 2.4.4. Phosphate Solubilization Assay

Bacteria were inoculated into tubes with 10 mL of Tryptone Soya Broth (TSB) medium (10%) for one week at 28 °C and shaken on an orbital shaker at 180 rpm. Cells were collected by centrifugation and washed twice with 0.8% NaCl solution, and 20 µL of this suspension was spotted on the National Botanical Research Institute’s phosphate growth medium (NBRIPM) containing, per 1 L, 15 g agar, 10 g glucose, 5 g Ca_3_(PO_4_)_2_, 5 g MgCl_2_·6H_2_O, 0.25 g MgSO_4_·7H_2_O, 0.2 g KCl, and 0.1 g (NH_4_)_2_SO_4_ [26]. The plates were incubated for 15 days at 25 °C. Positive phosphate solubilization was confirmed by the appearance of clear zones around spots.

#### 2.4.5. Siderophore Production

The isolates were selected for their ability to produce siderophores in the CAS medium [27]. Bacteria were collected from TBS, resuspended, and washed twice with phosphate-buffered saline pH 6.5. A 20 μL aliquot of bacterial suspension was spotted on CAS agar plates. The production of siderophores was checked daily for a color change from blue to red around each colony.

#### 2.4.6. Biocontrol Test

For the biocontrol test with the phytopathogenic fungus *Fusarium oxysporum* f. sp. *phaseoli*, mycelial discs of the fungus were collected and placed under a plate (1.5 cm) from the edge. On the opposite side of the plate, a streak of isolates was inoculated 1.5 cm from the edge. The plates were maintained at 25°C for one week. The percentage of growth inhibition was calculated using the formula (R1-R2)/R1 × 100, where R1 is the radial distance of the *F. oxysporum* f. sp. *phaseoli* mycelium in the absence of the antagonist from the center to the edge of the plate (measured in mm) and R2 is the growth distance of *F. oxysporum* f. sp. *phaseoli* from the center of the plate to the bank toward the isolate.

### 2.5. Plant Growth Promotion in Greenhouse Experiment

The seeds of soybean genotype Conquista were kindly provided by Thalita Avelar Monteiro from the Department of Plant Pathology of UFV. Seeds were surface-sterilized and inoculated with SOY2, SOY5, and SOY23 isolates by mixing for 2 h in the inoculum (10^8^ CFU mL^−1^ (DO550 = 0.1). A control treatment was achieved by mixing the seeds with sterilized saline solution (0.85%). The seedlings were grown in plastic trays containing 500 g of a mixture of soil, sand, and manure (3:2:1). The plants were grown under natural sunlight in a greenhouse with an average daytime temperature of 12 to 33 °C. Soybean plants were watered daily with the same volume until the first trifoliate (stage V1) leaves emerged, after which a water restriction treatment was imposed. The soybean plants were subjected to the following two water treatments: soil relative water content of 30% (control) and 5% (drought stress). The soil water levels were monitored daily. An evaluation was performed 30 d after sowing. The leaf area, number of nodes, shoot and root lengths, and shoot and root dry biomass were determined. The greenhouse experiments were conducted using a completely randomized design. The data were subjected to one-way ANOVA and the Skott–Knott clustering algorithm.

### 2.6. DNA Extraction and Whole-Genome Sequencing

*Kosakonia* sp. strain SOY2 and *Agrobacterium* sp. strain SOY23 were grown into a flask containing 20 mL of liquid DSMZ medium for one week at 28 °C and shaken on an orbital shaker at 180 rpm. Cells were collected by centrifugation and Genomic DNA was extracted using the Wizard^®^ Genomic DNA Purification Kit (Promega Corp., Madisonm, WI, USA.), as recommended by the manufacturer. DNA was checked for quality using a NanoDrop 2000 (Thermo Fisher Scientific, Waltham, MA, USA) and subjected to gel electrophoresis (0.8% of agarose). The whole genome was sequenced using the DNBseq Sequencing platform at BGI, Inc.

### 2.7. Genome Assembly

Raw data with adapter or low-quality sequences were filtered. We first went through a series of data processing to remove contamination and obtain valid data. This step was completed using the bynSOAPnuke software. SOAPnuke software filter parameters: “-n 0.01 -l 20 -q 0.4 -ada Mis 3 -out QualSys 1 -minReadLen 150” [28]. The genome was assembled using a de novo assembler implemented as an initial assembly graph from short reads in Unicycler [29], and the assembly metrics were evaluated using QUAST v4.6 [30]. The completeness and contamination of all MAGs were estimated using CheckM (v1.0.11) [31]. The assemblies were annotated using the Prokka v. 1.14.6 [32]. The genomes were submitted to the National Center for Biotechnology Information (NCBI) GenBank. The genome sequence data were uploaded to the Type (Strain) Genome Server (TYGS), a free bioinformatics platform available at https://tygs.dsmz.de for a whole genome-based taxonomic analysis [33].

### 2.8. Data Retrieving from a Public Database and Bioinformatics Analysis

A total of 169 genome sequences were retrieved from the NCBI database (last accessed in May 2020) (Table S1). These sequences were manually checked for their association with soil, plants, and rhizosphere according to the BioSample database. The proteome of the genomes was used to predict plant growth-promoting traits (PGPTs) through PLaBAse (v1.01, http://plabase.informatik.uni-tuebingen.de/pb/plabase.php) [34]. We also mined biosynthetic gene clusters (BGCs) using antiSMASH v5.1 [35]. Networks using similarity Minimum Information about a Biosynthetic Gene cluster database (MIBiG), using a locally installed version of the BiG-SCAPE software [36] with the local option enabled and a distance cut-off score of 0.3. The generated network was imported into Cytoscape version 3.7.2 and analyzed using default algorithms [37].

## 3. Results

### 3.1. The Selection of Distinct Rhizobacteria with the Potential for Novel Taxonomic Species

VL55 medium, an oligotrophic medium commonly employed for the selection of slow-growing bacteria, was used to isolate rhizobacteria. We strictly selected 22 isolates rhizobacterial colonies with morphologically distinct characteristics, including shape, color, size, and texture, from soybean soil samples collected in the experimental field (Figure 1A). The isolates were assigned the acronym SOY (soybean) followed by a sequential number. A DNA fingerprint analysis was performed to examine the genetic profiles of the isolates.

Three molecular markers were tested: ERIC, BOX, and GTG. The gel displayed various band patterns employing the markers, with BOX and GTG markers displaying more determinant characteristics for isolate identification (Figure S1). As a result, these two markers were chosen for the genetic profiling of the 22 isolates. A dendrogram revealed three large clusters for the BOX marker (Figure S2A) and three large clusters for the GTG marker (Figure S2B), each with several ramifications (Figure S2B). In general, most isolates had distinct genetic profiles; nevertheless, comparable profiles, such as isolated SOY19 and SOY20 for both markers, were detected, suggesting that the two isolates were genetically related (Figure S2). These findings suggest that this selection strategy allowed for the isolation of genetically distinct isolates.

Taxonomic identification of the isolates was based on sequencing of the gene encoding 16S rRNA. MEGAX was used to create a phylogenetic tree of the sequences. The retrieved sequences were compared with those in the NCBI database in terms of coverage and identity. Species closely related to ten genera were found: *Kosakonia, Microbacterium, Mycobacterium, Methylobacterium, Monashia*, *Novosphingobium*, *Pandoraea, Arthrobacter, Stenotrophomonas*, and *Rhizobium* (Table S2, Figure 1B). The 16S phylogenetic tree analysis also found ten groupings related to the genera. Thirteen of the 22 isolates analyzed had sequence identities lower than 96% when compared to the database sequences, indicating that these taxa may belong to new genera or species. This finding was supported by phylogenetic analysis, which showed that while the isolates were related to these taxa, different clades were formed (Figure 1B). Based on the comparison of isolates by NCBI and phylogeny, the proposed classification of these isolates is shown in Table S2.

### 3.2. The Novel Bacterial Species Exhibited Plant Growth-Promoting Traits

**Figure 2:**
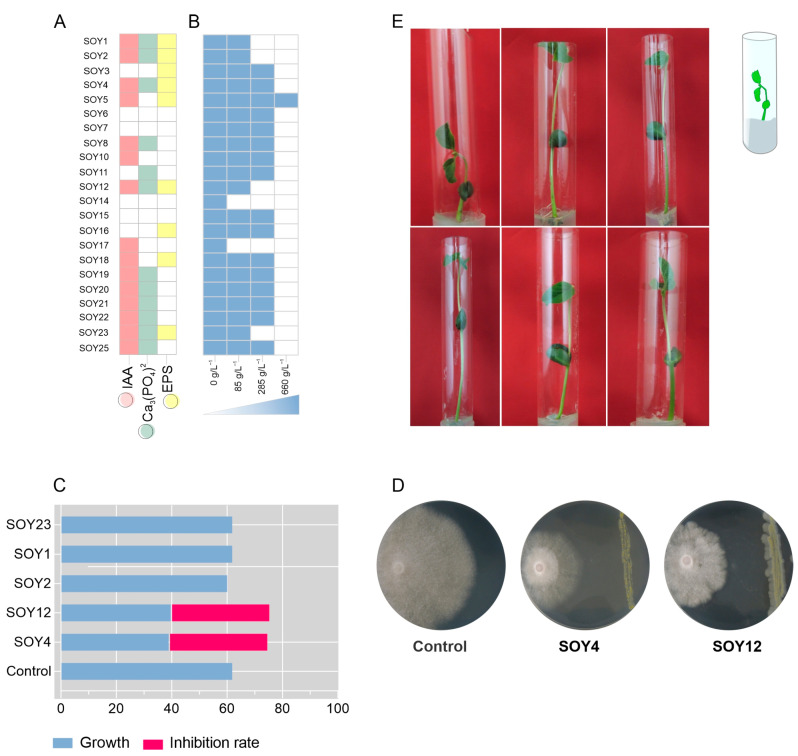
In vitro features for plants promoting growth and biological control. (**A**) The production of features for plants promotes growth. Left to right: synthesis of indole acetic acid (IAA) in pink; phosphate solubilization (Ca_3_(PO_4_)_2_ in green; exopolysaccharide (EPS) production in yellow. Colored squares indicate the growth of each isolate. (**B**) Ability of bacteria to grow in a medium with reduced water availability containing different sorbitol concentrations at increasing levels. The colored squads indicate the growth of each isolate. (**C**) Biological phytopathogen control. On the x-axis, the growth rate in centimeters by the fungus and the isolates are plotted on the y-axis. The growth of the fungus is shown in blue, and the inhibition rate of the fungal growth is shown in pink. (**D**) Paired culture test with *Microbacterium* sp. strain SOY4 and *Monashia* sp. strain SOY12 against *Fusarium oxy*sp*orum* f. sp. *phaseoli* sp. (**E**) In vitro soybean growth-promotion tests; left to right: control plant with PBS buffer, plant inoculated with SOY1, plants inoculated with SOY2, SOY4, and SOY12 plants inoculated with SOY23.

The isolates were analyzed for their capacity to generate growth-promoting characteristics such as IAA, EPS, and phosphate solubilization. Seventy-two percent (*n* = 16) of the 22 rhizobacterial isolates produced IAA, 68% (*n* = 12) solubilized phosphate, and 45% (*n* = 10) produced EPS (Figure 2A). Furthermore, the isolates were tested for their ability to grow in a medium with low water activity. DSMZ medium containing four amounts of sorbitol (0 g/L^−1^, 285 g/L^−1^, 520 g/L^−1^, and 660 g/L^−1^) was used (Figure 2B). Approximately 91% (*n* = 22) showed positive growth at 285 g/L^- 1^ sorbitol concentration, 75% (*n* = 18) showed positive growth at 520 g/L^−1^ sorbitol concentration, and only *Microbacterium* sp. strain SOY5 exhibited positive growth at all sorbitol concentrations. The lower the water activity, the higher the sorbitol content. Therefore, we observed that bacterial growth was minimal at the highest concentration of sorbitol. Taken together, these findings suggest that the majority of the isolates possessed one or more growth-promoting properties and that these bacteria may enhance plant development under water stress.

Five isolates with positive results for IAA, phosphate solubilization, EPS, and growth in reduced water were selected: *Kosakonia* sp. strain SOY1, *Kosakonia* sp. strain SOY2, *Microbacterium* sp. strain SOY4, *Monashia* sp. strain SOY12, and *Agrobacterium* sp. strain SOY23. The isolates were initially evaluated to determine their growth pattern over time (in hours) for the plant test. The bacteria were grown for 10 h, during which time a typical growth curve was observed, with the bacteria reaching the initial stationary phase (Figure S3). After 3 h of incubation, an aliquot of the culture medium at OD 1.0 was plated on a nutritional agar medium for cell counting and viability analysis. The plate cell count for all isolates showed a cell density of 10^8^ CFU/mL, thus validating the optimal spot on the growth curve for plant inoculation. Soybean seeds were treated with the isolates and stored for two weeks. Plants treated with the bacterial suspensions of the isolates grew at a faster rate at the end of the first week. Given that the initial leaflets were visible in all treatments, a rise in the stems of the plants treated with bacterial suspensions was generally observed under laboratory conditions.

Finally, the five isolates previously chosen for growth promotion were examined for biocontrol efficacy against the phytopathogen *Fusarium oxy*sp*orum* f. sp. *phaseoli*, which causes plant wilting in common beans [38]. *Fusarium oxy*sp*orum* f. sp. *phaseoli* was chosen as the model pathogen because *F. oxy*sp*orum* is a globally dispersed disease with several hosts. Paired in vitro culture activity research revealed that *Microbacterium* sp. strain SOY4 and *Monashia* sp. strain SOY12 inhibited plant pathogen development by 35%, indicating that these two isolates also have the ability to control phytopathogens.

### 3.3. Two Isolates Showed the Ability to Enhance Soybean Growth under Drought in Greenhouse Conditions

**Figure 3:**
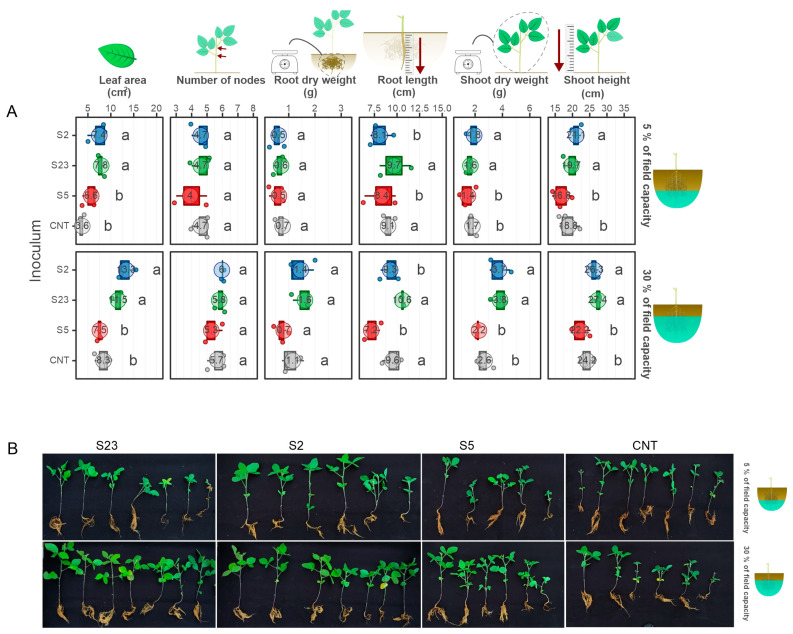
Soybean growth under drought in greenhouse conditions. (**A**) Phenotypic traits were measured in soybean plants under two water deficit conditions (5% and 30% field capacity) and inoculation with three bacterial isolates. Treatments that showed the same lowercase letter within each water deficit condition did not show statistically significant differences according to the Skott –Knott test. (**B**) Non-inoculated soybean plants (control) and plants inoculated with different bacteria (SOY2, SOY5, and SOY23) and under drought stress.

The ability to enhance soybean growth under drought in greenhouse conditions was tested for the *Kosakonia* sp. strain SOY2, *Microbacterium* sp. strain SOY5, and *Agrobacterium* sp. strain SOY23. We found that the SOY2 and SOY23 bacteria showed potential in water stress mitigation. Under both water stress conditions, plants whose seeds were inoculated with these bacteria generated more dry matter in the shoot (Figure 3) and had a smaller reduction in leaf area than the other treatments (control without inoculation and inoculation with SOY5) (severe and moderate). There was no statistically significant change in the root system mass across treatments (Figure 3A). Plants inoculated with these two bacteria (SOY2 and SOY23) had a shorter root length than the other treatments. Under water stress, the plant’s root system usually changes, such as the production of more sharp angles between, to make it deeper [39], allowing the plant to use the water available in deeper soil strata. However, changing the architecture of the root system consumes energy that the plant may otherwise employ. Thus, when compared to SOY2 and SOY23, the increase in root length in SOY5 and the control treatments may imply less stress mitigation.

Non-inoculated plants showed a 56% reduction in leaf area (Figure 3A), whereas SOY2 and SOY23 bacterial treatments reduced leaf area by 25 and 32%, respectively. In other words, compared to the control, inoculation with these bacteria reduced the leaf area loss by more than half. Plants with larger leaf areas have a larger surface area for catching light, which implies that photo-assimilates (sugars) are produced at a higher rate than plants with smaller leaf areas, allowing for more grain filling during the reproductive phase (Figure 3B).

### 3.4. The Genomic Sequences of Kosakonia and Agrobacterium

**Figure 4:**
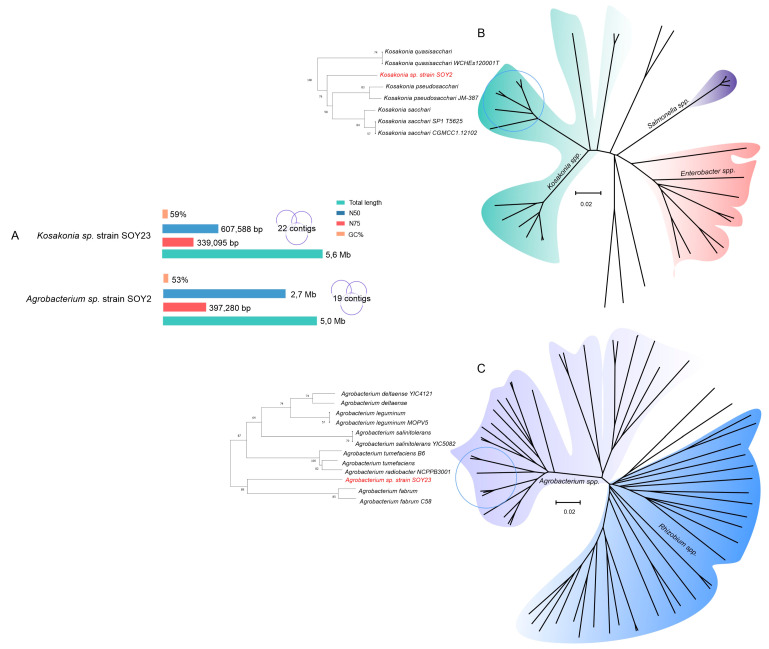
Whole-genome sequences of SOY2 and SOY23 rhizobacteria. (**A**) Genomic features of *Kosakonia* sp. strain SOY2 and *Agrobacterium* sp. SOY23. (**B**) Phylogenomic tree of *Kosakonia* sp. strain SOY2 inferred with FastME 2.1.6.1 from GBDP distances calculated from genome sequences. (**C**) Phylogenomic tree of *Agrobacterium* sp. strain SOY23. The branch lengths were scaled in terms of the GBDP distance formula d_5_. The numbers above are GBDP pseudo-bootstrap support values of >60 % from 100 replications, with an average branch support of 80.8 %. The tree was rooted at its midpoint.

Given the potential of *Kosakonia* sp. strain SOY2 and *Agrobacterium* sp. strain SOY23 to positive interact with soybean in vitro and in planta, here we report a draft genome sequence of these two strains to gain a better understanding of this interaction with the plant. The assembly of *Kosakonia* sp. strain SOY2 consisted of 19 contigs with a total sequence length of 5,035,089 bp, an N50 value of 2,707,390 bp, and a GC content of 53.81% (Figure 4A). The closest placement taxonomy result was *Kosakonia* sp000410515, with an average nucleotide identity (ANI) of 95.63 %. According to the whole-genome phylogeny, this strain is related to plant-associated *Kosakonia* spp. However, the genome was not assigned to any species (Figure 4B). A total of 4748 CDS, 3 rRNAs (5S, 16S, and 23S), 71 tRNAs, and 1 tmRNA were found in the *Kosakonia* sp. strain SOY2 genome.

The assembly of *Agrobacterium* sp. strain SOY23 consisted of 22 contigs with a total sequence length of 5,626,101 bp, N50 value of 607,588 bp, and GC content of 59.59%. Surprisingly, the genome was not assigned to any closest species, as it was outside the predefined ANI radius (Table S2, Figure 4B). CheckM was employed to estimate the completeness and contamination of the genome, which were 99.94% and 0.35%, respectively. 

A total of 5225 CDS, 46 tRNAs, and 1 tmRNA were found in the *Agrobacterium* sp. strain SOY23 genome.

### 3.5. A Genome Mining Analysis of Novel Species of Soil Bacteria Revealed Several Proteins with Traits That Promote Plant Growth

**Figure 5:**
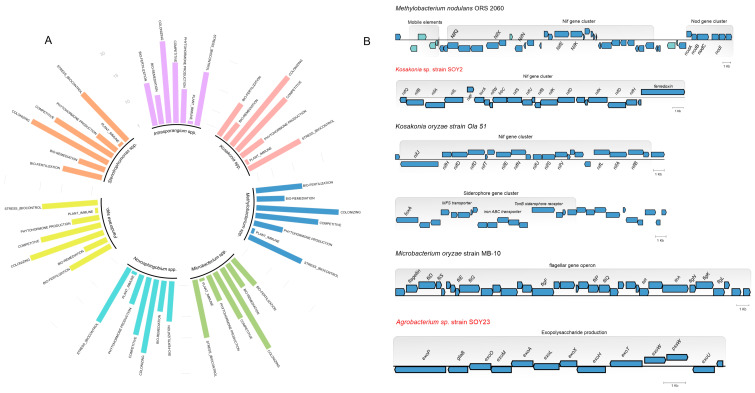
In silico identification and comparison of plant growth-promoting genes. (**A**) Circular plot illustrating the average number of plant growth-promoting genes found in each of the seven genera. (**B**) Genes encoding plant colonization and plant growth promotion associated with nitrogen fixation, siderophores, flagella, and exopolysaccharides found in the genomes.

To better understand the potential of all species described here, we gathered publicly available genomes from the NCBI database and performed in silico identification and comparison of plant growth-promoting genes. We first sought plant growth-promoting genes in 169 genome sequences associated with soil, plants, and the rhizosphere (Table S1). We found that the majority of these genomes encode proteins with direct effects on the plant, such as biofertilization, iron acquisition, P and K solubilization, phytohormone synthesis, and nitrogen fixation. These genomes possess heavy metal resistance genes such as cobalt, copper, nickel, and selenium, which might be exploited for bioremediation. Furthermore, these bacteria encode traits that aid in plant system colonization, such as chemotaxis proteins and motility, and they may utilize plant-derived amino acids, sugars, and peptides. In addition, it neutralizes abiotic stresses, such as high and low temperature and acidic, osmotic, and salinity stress. The average number of each gene class per genome is shown in Figure 5 and Table S3.

An essential operon encoding plant growth-promoting gene was found. The *nif* gene cluster in *Methylobacterium nodulans* shows the *nif* cluster surrounded by mobile genetic elements and nod genes organized in a single operon with six ORFs. We also found a conservative nif operon in *Kosakonia* sp. strain SOY2 and *K. oryzae*, as well as a single operon for siderophore synthesis. In addition, a flagellar operon observed in *M. oryzae* responsible for plant system colonization, and a single cluster for EPS synthesis in *Agrobacterium* sp. strain SOY23, were also mapped.

**Figure 6:**
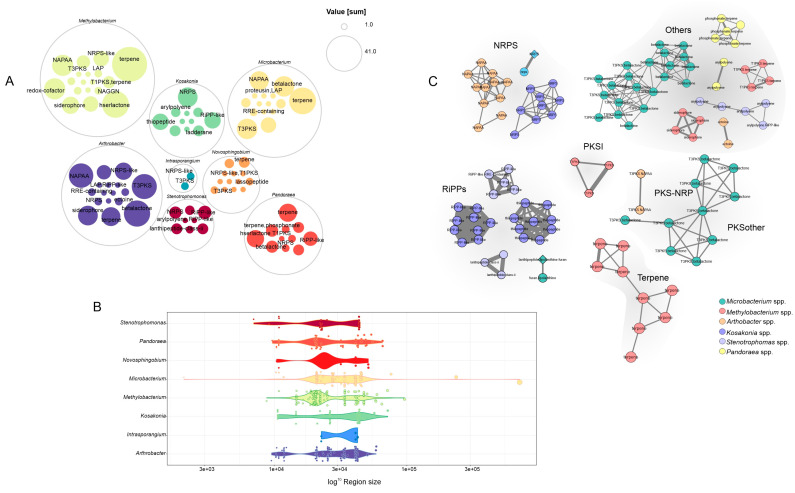
Identification and comparison of biosynthetic gene clusters. (**A**) Genera-level BGCs distribution. (**B**) Length of BGCs across genera. (**C**) Sequence similarity networks of the BGCs.

We also explored the repertoire of secondary metabolite biosynthetic gene clusters (BGCs) encoded within these genomes using AntiSMASH. In total, 506 putative BGC regions were identified (Table S4). The majority of the BGC were found in the *Methylobacterium* (146) followed by *Arthrobacter* (120) (Figure 6A). There were 93 terpene-containing gene clusters found, mostly in *Methylobacterium*, 47 NAPAA clusters from three genera (*Arthrobacter*, *Methylobacterium*, and *Microbacterium*), 43 betalactone clusters from 76 phyla, and 40 Type III polyketide synthases clusters. In general, the BGC regions in length varied from 2 kb to 76 kb, the largest size an NRP + Polyketide: Modular type I found in the *Microbacterium amylolyticum* DSM 24221 (Figure 6B). In addition, we used BiG-SCAPE to construct a BGC sequence similarity network (Figure 6C). We discovered that the majority of BGCs were taxonomically distributed among the same genera and architecturally heterogeneous (Figures S4 and S5). In general, nearly none of the BGCs found here were grouped with the MIBiG reference BGCs, indicating a repertoire of novel secondary metabolite BGCs (Figure 6C).

## 4. Discussion

In this study, 22 soybean growth-promoting rhizobacteria were identified. A total of 78% of the 22 bacteria isolated exhibited positive results for water stress growth, indicating their potential for application in crops with a shortage of water. The isolates produced IAA, the primary auxin found in plants, which is synthesized in the apical system of the stem and transported to the roots; plant-associated microbes can also synthesize it. Its primary effect is the growth of roots and stems [40,41]. An in vitro test of soybean revealed this elongation effect. Furthermore, the isolates were able to solubilize phosphorus, an important element in plant metabolism. Plant growth is hampered by its absence; nevertheless, it is the least available nutrient for the plant, since it is held by the precipitation of other soil elements, resulting in insoluble inorganic phosphates with the lowest available for the plant [42,43]. As a result, bacteria with the capacity to solubilize insoluble inorganic phosphate sources, increasing the soluble phosphorus level in the soil solution, and plant availability, play a crucial role in the phosphorus biogeochemical cycle [44].

The genera *Bacillus*, *Pseudomonas*, and *Burkholderia* have been commonly identified as widely prevalent growth-promoting bacteria in soybeans [45,46,47]. Here, by contrast, we discovered *Kosakania* sp., *Microbacterium* sp., *Mycobacterium* sp., *Methylobacterium* sp., *Novosphingobium* sp., *Anthrobacter* sp., *Stenotrophomonas* sp., *Monashia* sp., and *Pandoraea* sp. This could be due to the VL55 isolation medium used in this study. VL55 is a defined medium that is low in nutrients and has pH in the most acidic range. Because xylan is the only available carbon source, it is commonly employed for the selection of bacteria that were previously classified as “unculturable” in soil [11,48]. Although the isolates identified in this study were grouped close to the aforementioned genera, phylogenetic analysis revealed that they may belong to new genera and/or species.

Some of the genera shown here are related to plant growth promotion activities, such as *Microbacterium* in neem growth promotion [49]. Another example is the genus *Methylobacterium*, which absorbs carbon molecules during endophytic associations and releases biopolymers, organic acids, coenzymes, vitamins, and toxins that aid in disease management [50,51]. Yet, few studies have been conducted to investigate the interactions of this genus with plants. In addition to species already known to promote plant growth, genera such as *Novosphingobium* are known to produce enzymes capable of degrading aromatic compounds [52,53]; *Arthrobacter* is used in many industrial applications and has the potential to be used in bioremediation [54]. Surprisingly, genera known to cause diseases in humans, such as *Pandoraea* and *Stenotrophomonas*, were isolated. However, based on the phylogenetic distance between the species, these isolates may belong to a distinct group of bacteria unrelated to human diseases.

*Microbacterium* sp. strain SOY4 and *Monashia* sp. strain SOY12 were effective in suppressing the phytopathogen *Fusarium oxysporum* f. sp. *phaseoli*. Both isolates were Actinobacteria, a phylum recognized for producing secondary metabolic metabolites with broad antifungal properties [55]. In addition, we have showed the ability of two isolates SOY2 (*Kosakonia* sp.) and SOY23 (*Agrobacterium* sp.) to enhance soybean growth under drought in greenhouse conditions. Plants seeds inoculated with these bacteria generated more dry matter in the shoots and had a smaller reduction in leaf area than the other treatments. Although *Agrobacterium* has been identified as a plant pathogenic bacterium, certain studies have shown that specific tumor-inducing *Agrobacterium* strains can stimulate plant growth in non-susceptible plant hosts [56,57].

The use of genomics techniques in investigations aimed at the potential of plant growth-promoting bacteria has provided evidence of the genetic characteristics that promote the microbe–plant interaction [58]. Here, we gained insight into the genomic potential of two bacteria of our isolates SOY2 and SOY23 along with the bacteria genus mentioned in this study. We confirmed that SOY2 and SOY23 did not belong to any of the nearest species and represented two novel bacteria species, and together with 169 genomes, they have a repertoire of genes encoding for plant growth-promoting proteins with direct effects on the plant, such as bio-fertilization, iron acquisition, P and K solubilization, phytohormone synthesis, and nitrogen fixation. In addition, secondary-metabolite BGCs analysis revealed a variety of novel secondary metabolite BGCs to be explored.

Well-known plant growth-promoting rhizobacteria (PGPR) have been widely used in the commercial sector with success; however, the search for new microbes that are resilient to adverse effects such as drought and have the potential to promote the growth of crops plants is becoming increasingly relevant, given climate change and its impact on food production. Chauhan and colleagues [59] reported several novel PGPRs that have not yet been achieved for commercial scales of production.

## 5. Conclusions

This work adds to the inclusion of new species with significant potential for promoting plant growth. Taken together, here we demonstrated novel soil bacteria with a growth-promoting capability that had not previously been reported for soybean. In addition, we demonstrated the importance of coupling a more complex medium culture with bioinformatics approaches to select new PGPR. The findings enable the identification of distinct bacteria with a high potential for promoting plant growth, opening the path for future research and uses in agriculture intending to reduce the environmental impact of synthetic industrial pesticides and fertilizers, and to help mitigate drought stress.

## Data Availability

In total, 16S rRNA sequences are available under the following accession numbers from OQ054593 to OQ054611 in the Center for Biotechnology Information (NCBI). The genomes sequences are available in the NCBI database under the accession number JAPVXH000000000 for *Kosakonia* sp. strain SOY2 and JAPWDT000000000 for *Agrobacterium* sp. strain SOY23. The accession numbers of publicly genome sequences used can be found in the Supplementary Material for this manuscript.

## Supplementary Materials

The following supporting information can be downloaded at: https://www.mdpi.com/article/10.3390/microorganisms11020300/s1, Figure S1. Depicts a Molecular Marker Test. According to the line above, the markers employed are delimiting the wells. M stands for GeneRuler 1 Kb Plus DNA Ladder molecular weight marker; Figure S2. Dendrogram analysis of isolate genetic profiles. The closeness value is shown by the colored circles with values at the intersections of the trees. (A) Dendrogram made use of a BOX marker. (B) Dendrogram with the GTG marker. The appropriate 1.5% agarose electrophoresis gels are shown below the dendrograms. The name of the isolate is used to denote each well. The 1 Kb and 100 bp markers are in the first and last wells, respectively; Figure S3. Standard growth curve for isolates. (A) Dispersion curve. The x-axis shows time in hours, and the y-axis shows absorbance in nm. (B) Bar graph depicting plaque growth after 3 h. The X-axis represents isolates, the y-axis represents colony-forming units (CFU) per ml; Figure S4. Biosynthetic gene cluster types identified from the 169 publicly genomic sequences available for soil bacteria; Figure S5. CORASON phylogeny of Biosynthetic gene cluster types identified from the 169 publicly genomic sequences available for soil bacteria; Table S1: Information regarding the 169 publicly available genomes obtained from NCBI database; Table S2: Taxonomic identification of isolates; Table S3: Classes of plant growth-promoting genes found in each of the genomes; Table S4: Biosynthetic gene clusters identified from the 169 publicly genomic sequences available for soil bacteria.

## References

[1] Berg G., Grube M., Schloter M., Smalla K. (2014). Unraveling the plant microbiome: Looking back and future perspectives. Front. Microbiol..

[2] Dastogeer K.M.G., Tumpa F.H., Sultana A., Akter M.A., Chakraborty A. (2020). Plant microbiome–an account of the factors that shape community composition and diversity. Curr. Plant Biol..

[3] Naylor D., Coleman-Derr D. (2018). Drought stress and root-associated bacterial communities. Front. Plant Sci..

[4] Staley J.T., Konopka A. (1985). Measurement of in situ activities of nonphotosynthetic microorganisms in aquatic and ter-restrial habitats. Annu. Rev. Microbiol..

[5] Bardgett R.D., van der Putten W.H. (2014). Belowground biodiversity and ecosystem functioning. Nature.

[6] Tiedje J.M., Asuming-Brempong S., Nüsslein K., Marsh T.L., Flynn S.J. (1999). Opening the black box of soil microbial di-versity. Appl. Soil Ecol..

[7] Sokol N.W., Slessarev E., Marschmann G.L., Nicolas A., Blazewicz S.J., Brodie E.L., Firestone M.K., Foley M.M., Hestrin R., Hungate B.A. (2022). Life and death in the soil microbiome: How ecological processes influence biogeo-chemistry. Nat. Rev. Microbiol..

[8] Andrews J.H., Harris R.F., Marshall K.C. (1986). R- and K-Selection and Microbial Ecology BT—Advances in Microbial Ecology.

[9] Kielak A.M., Cipriano M.A.P., Kuramae E.E. (2016). Acidobacteria strains from subdivision 1 act as plant growth-promoting bacteria. Arch. Microbiol..

[10] Huang Y.S., Shen F.T. (2016). Bioprospecting of facultatively oligotrophic bacteria from non-rhizospheric soils. Appl. Soil Ecol..

[11] Joseph S.J., Hugenholtz P., Sangwan P., Osborne C.A., Janssen P.H. (2003). Laboratory cultivation of widespread and previously uncultured soil bacteria. Appl. Environ. Microbiol..

[12] Kato S., Yamagishi A., Daimon S., Kawasaki K., Tamaki H., Kitagawa W., Abe A., Tanaka M., Sone T., Asano K. (2018). Isolation of previously uncultured slow-growing bacteria by using a simple modification in the preparation of agar media. Appl. Environ. Microbiol..

[13] Pulschen A.A., Bendia A.G., Fricker A.D., Pellizari V.H., Galante D., Rodrigues F. (2017). Isolation of uncultured bacteria from antarctica using long incubation periods and low nutritional media. Front. Microbiol..

[14] Tschech A., Pfennig N. (1984). Growth yield increase linked to caffeate reduction in Acetobacterium Woodii. Arch. Microbiol..

[15] Widdel F., Kohring G.-W., Mayer F. (1983). Studies on dissimilatory sulfate-reducing bacteria that decompose fatty acids. Arch. Microbiol..

[16] Koeuth T., Versalovic J., Lupski J.R. (1995). Differential subsequence conservation of interspersed repetitive Streptococcus Pneumoniae BOX elements in diverse bacteria. Genome Res..

[17] Versalovic J., Koeuth T., Lupski J.R. (1991). Distribution of repetitive dna sequences in eubacteria and application to fin-gerprinting of bacterial genomes. Nucleic Acids Res..

[18] Heuer H., Krsek M., Baker P., Smalla K., Wellington E.M. (1997). Analysis of Actinomycete communities by specific am-plification of genes encoding 16S rRNA and gel-electrophoretic separation in denaturing gradients. Appl. Environ. Microbiol..

[19] Altschul S.F., Gish W., Miller W., Myers E.W., Lipman D.J. (1990). Basic Local Alignment Search Tool. J. Mol. Biol..

[20] Katoh K., Rozewicki J., Yamada K.D. (2019). MAFFT Online Service: Multiple Sequence Alignment, Interactive Sequence Choice and Visualization. Brief Bioinform..

[21] Larkin M.A., Blackshields G., Brown N.P., Chenna R., McGettigan P.A., McWilliam H., Valentin F., Wallace I.M., Wilm A., Lopez R. (2007). Clustal W and Clustal X version 2.0. Bioinformatics.

[22] Tamura K., Stecher G., Kumar S. (2021). MEGA11: Molecular Evolutionary Genetics Analysis Version 11. Mol. Biol. Evol..

[23] Kavamura V.N., Santos S.N., da Silva J.L., Parma M.M., Ávila L.A., Visconti A., Zucchi T.D., Taketani R.G., Andreote F.D., Melo I.S. (2013). de Screening of Brazilian cacti rhizobacteria for plant growth promotion under drought. Microbiol Res.

[24] Paulo E.M., Vasconcelos M.P., Oliveira I.S., de Affe H.M.J., Nascimento R., de Melo I.S., de Roque M.R.A., de Assis S.A. (2012). An Alternative method for screening lactic acid bacteria for the production of exopolysaccharides with rapid confirmation. Food Sci. Technol..

[25] Gordon S.A., Weber R.P. (1951). Colorimetric estimation of indoleacetic acid. Plant Physiol..

[26] Nautiyal C.S. (1999). An efficient microbiological growth medium for screening phosphate solubilizing Microorganisms. FEMS Microbiol Lett..

[27] Schwyn B., Neilands J.B. (1987). Universal chemical assay for the detection and determination of siderophores. Anal. Biochem..

[28] Chan Y., Chen Y., Shi C., Huang Z., Yong Z., Shengkang L., Li Y., Ye J., Yu C., Li Z. (2017). SOAPnuke: A mapreduce acceleration supported software for integrated quality control and preprocessing of high-throughput se-quencing data. Gigascience.

[29] Wick R.R., Judd L.M., Gorrie C.L., Holt K.E. (2017). Unicycler: Resolving bacterial genome assemblies from short and long sequencing reads. PLoS Comput. Biol..

[30] Gurevich A., Saveliev V., Vyahhi N., Tesler G. (2013). QUAST: Quality Assessment Tool for Genome Assemblies. Bioinfor-matics..

[31] Parks D., Imelfort M., Skennerton C., Philip H., Tyson G. (2015). CheckM: Assessing the Quality of Microbial Genomes Recovered from Isolates, Single Cells, and Metagenomes. Genome Res..

[32] Seemann T. (2014). Prokka: Rapid Prokaryotic Genome Annotation. Bioinformatics.

[33] Meier-Kolthoff J.P., Göker M. (2019). TYGS Is an automated high-throughput platform for state-of-the-art genome-based taxonomy. Nat. Commun..

[34] Patz S., Gautam A., Becker M., Ruppel S., Rodríguez-Palenzuela P., Huson D.H. (2021). PLaBAse: A comprehensive web resource for analyzing the plant growth-promoting potential of plant-associated bacteria. bioRxiv.

[35] Blin K., Shaw S., Steinke K., Villebro R., Ziemert N., Lee S.Y., Medema M.H., Weber T. (2019). AntiSMASH 5.0: Updates to the secondary metabolite genome mining pipeline. Nucleic Acids Res..

[36] Navarro-Munoz J., Selem N., Mullowney M., Kautsar S., Tryon J., Parkinson E., de los Santos E.L., Yeong M., Cruz-Morales P., Abubucker S. (2020). A computational framework for systematic exploration of biosynthetic diversity from large-scale genomic data. Nat. Chem. Biol..

[37] Shannon P., Markiel A., Ozier O., Baliga N.S., Wang J.T., Ramage D., Amin N., Schwikowski B., Ideker T. (2003). Cy-toscape: A software environment for integrated models of biomolecular interaction networks. Genome Res.

[38] Abawi G.S., Pastor Corrales M.A., Centro Internacional de Agricultura Tropical (1990). Root Rots of Beans in Latin America and Africa: Diagnosis, Research Methodologies, and Management Strategies.

[39] Fenta B.A., Beebe S.E., Kunert K.J., Burridge J.D., Barlow K.M., Lynch J.P., Foyer C.H. (2014). Field phenotyping of soy-bean roots for drought stress tolerance. Agronomy.

[40] Glick B.R. (2012). Plant Growth-Promoting Bacteria: Mechanisms and Applications. Scientifica (Cairo).

[41] Patten C.L., Glick B.R. (1996). Bacterial Biosynthesis of Indole-3-Acetic Acid. Can. J. Microbiol..

[42] Ehrlich H.L. (1998). Geomicrobiology: Its Significance for Geology. Earth Sci. Rev..

[43] Hayat R., Ali S., Amara U., Khalid R., Ahmed I. (2010). Soil Beneficial Bacteria and Their Role in Plant Growth Promotion: A Review. Ann. Microbiol..

[44] Yang X., Post W.M., Thornton P.E., Jain A. (2013). The Distribution of Soil Phosphorus for Global Biogeochemical Modeling. Biogeosciences.

[45] Kuklinsky-Sobral J., Araújo W.L., Mendes R., Geraldi I.O., Pizzirani-Kleiner A.A., Azevedo J.L. (2004). Isolation and characterization of soybean-associated bacteria and their potential for plant growth promotion. Environ. Microbiol..

[46] Moretti L.G., Crusciol C.A.C., Kuramae E.E., Bossolani J.W., Moreira A., Costa N.R., Alves C.J., Pascoaloto I.M., Rondina A.B.L., Hungria M. (2020). Effects of Growth-Promoting Bacteria on Soybean Root Activity, Plant Development, and Yield. Agron. J..

[47] Schmidt J., Messmer M., Wilbois K.-P. (2015). Beneficial Microorganisms for Soybean (Glycine max (L.) Merr), with a Focus on Low Root-Zone Temperatures. Plant Soil.

[48] Sait M., Hugenholtz P., Janssen P.H. (2002). Cultivation of Globally Distributed Soil Bacteria from Phylogenetic Lineages Previously Only Detected in Cultivation-Independent Surveys. Environ. Microbiol..

[49] Madhaiyan M., Poonguzhali S., Lee J.-S., Lee K.-C., Saravanan V.S., Santhanakrishnan P. (2010). Microbacterium aza-dirachtae Sp. Nov., a Plant-Growth-Promoting Actinobacterium Isolated from the Rhizoplane of Neem Seedlings. Int. J. Syst. Evol. Microbiol..

[50] Dourado M.N., Bogas A.C., Pomini A.M., Andreote F.D., Quecine M.C., Marsaioli A.J., Araújo W.L. (2013). Methylobac-terium-Plant Interaction Genes Regulated by Plant Exudate and Quorum Sensing Molecules. Braz. J. Microbiol..

[51] Grossi C.E.M., Fantino E., Serral F., Zawoznik M.S., Fernandez Do Porto D.A., Ulloa R.M. (2020). Methylobacterium Sp. 2A Is a Plant Growth-Promoting Rhizobacteria That Has the Potential to Improve Potato Crop Yield Under Adverse Conditions. Front. Plant Sci..

[52] Liu Z.-P., Wang B.-J., Liu Y.-H., Liu S.-J. (2005). *Novosphingobium* Taihuense sp. nov., a Novel Aro-matic-Compound-Degrading Bacterium Isolated from Taihu Lake, China. Int. J. Syst. Evol. Microbiol..

[53] Sohn J.H., Kwon K.K., Kang J.-H., Jung H.-B., Kim S.-J. (2004). *Novosphingobium* Pentaromativorans sp. nov., a High-Molecular-Mass Polycyclic Aromatic Hydrocarbon-Degrading Bacterium Isolated from Estuarine Sediment. Int. J. Syst. Evol. Microbiol..

[54] Westerberg K., Elväng A.M., Stackebrandt E., Jansson J.K. (2000). Arthrobacter chlorophenolicus sp. nov., a New Species Capable of Degrading High Concentrations of 4-Chlorophenol. Int. J. Syst. Evol. Microbiol..

[55] Salwan R., Sharma V. (2020). Molecular and biotechnological aspects of secondary metabolites in actinobacteria. Microbiol. Res..

[56] Bruto M., Prigent-Combaret C., Muller D., Moënne-Loccoz Y. (2014). Analysis of Genes Contributing to Plant-Beneficial Functions in Plant Growth-Promoting Rhizobacteria and Related Proteobacteria. Sci. Rep..

[57] Walker V., Bruto M., Bellvert F., Bally R., Muller D., Prigent-Combaret C., Moënne-Loccoz Y., Comte G. (2013). Unex-pected Phytostimulatory Behavior for Escherichia coli and Agrobacterium Tumefaciens Model Strains. Mol. Plant Microbe Interact..

[58] Imam J., Singh P.K., Shukla P. (2016). Plant Microbe Interactions in Post Genomic Era: Perspectives and Applications. Front. Microbiol..

[59] Chauhan H., Bagyaraj D.J., Selvakumar G., Sundaram S.P. (2015). Novel Plant Growth Promoting Rhizobacte-ria—Prospects and Potential. Appl. Soil Ecol..

