# Supplementary material for: Harnessing Novel Soil Bacteria for Beneficial Interactions with Soybean
Source: Microorganisms. 2023 Jan 23;11(2):300. doi: 10.3390/microorganisms11020300 (PMC9964534; doi:10.3390/microorganisms11020300)
Supplement: Supplementary file 1 [file microorganisms-11-00300-s001.zip › microorganisms-2136508-Suppl. conv fig.pdf]

Supplementary materials

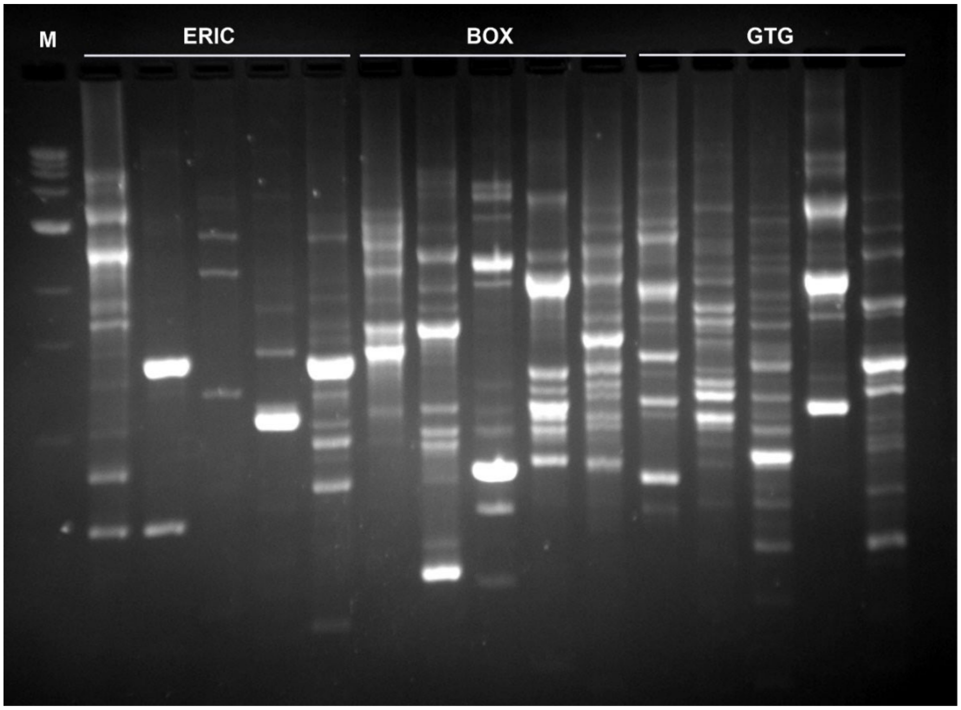

Figure S1. Depicts a Molecular Marker Test. According to the line above, the markers employed are delimiting the wells. M stands for GeneRuler 1Kb Plus DNA Ladder molecular weight marker.

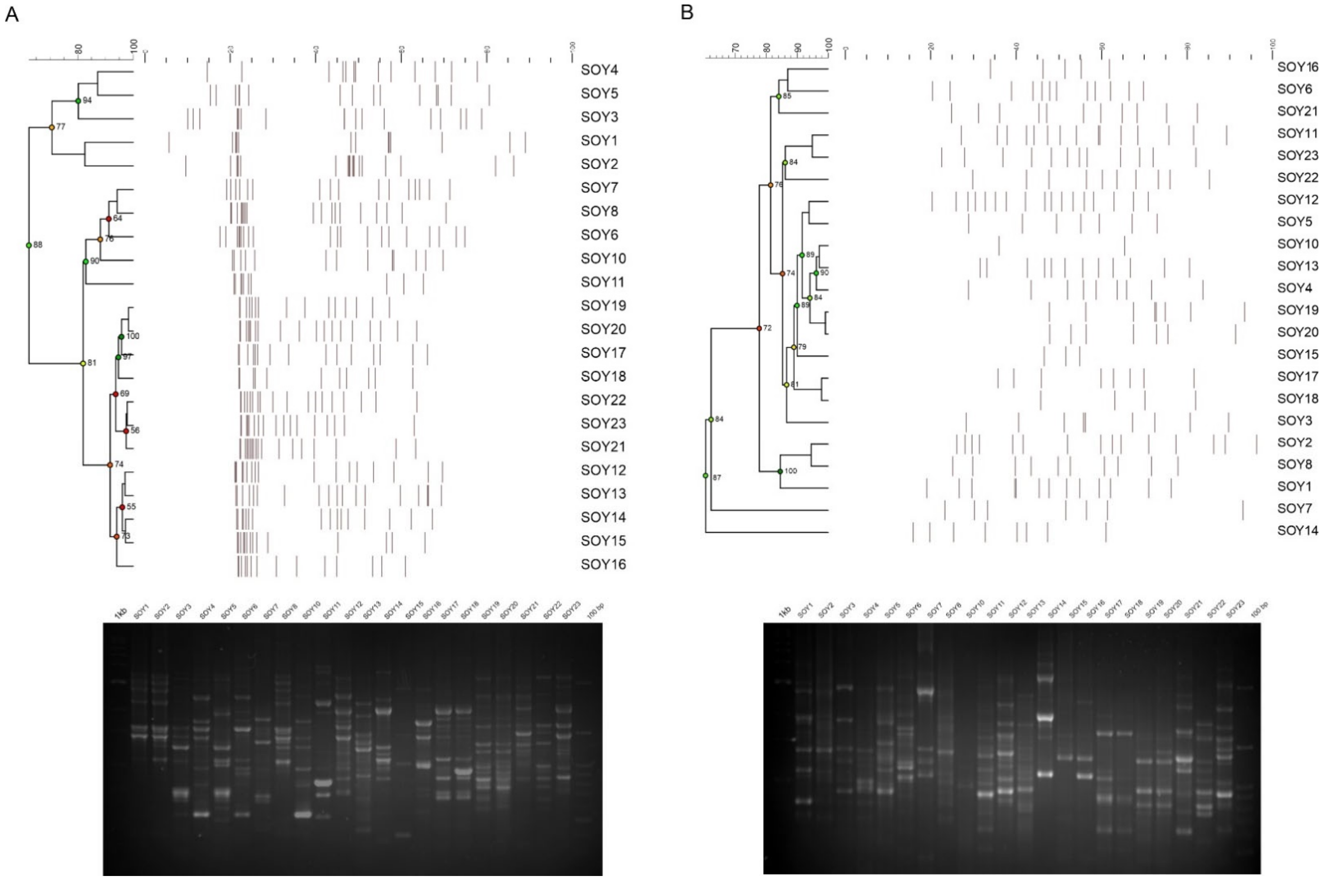

Figure S2. Dendrogram analysis of isolate genetic profiles. The closeness value is shown by the colored circles with values at the intersections of the trees. **A**). Dendrogram made use of a BOX marker. **B**). dendrogram with the GTG marker. The appropriate 1.5% agarose electrophoresis gels are shown below the dendrograms. The name of the isolate is used to denote each well. The 1Kb and 100pb markers are in the first and last wells, respectively.

A

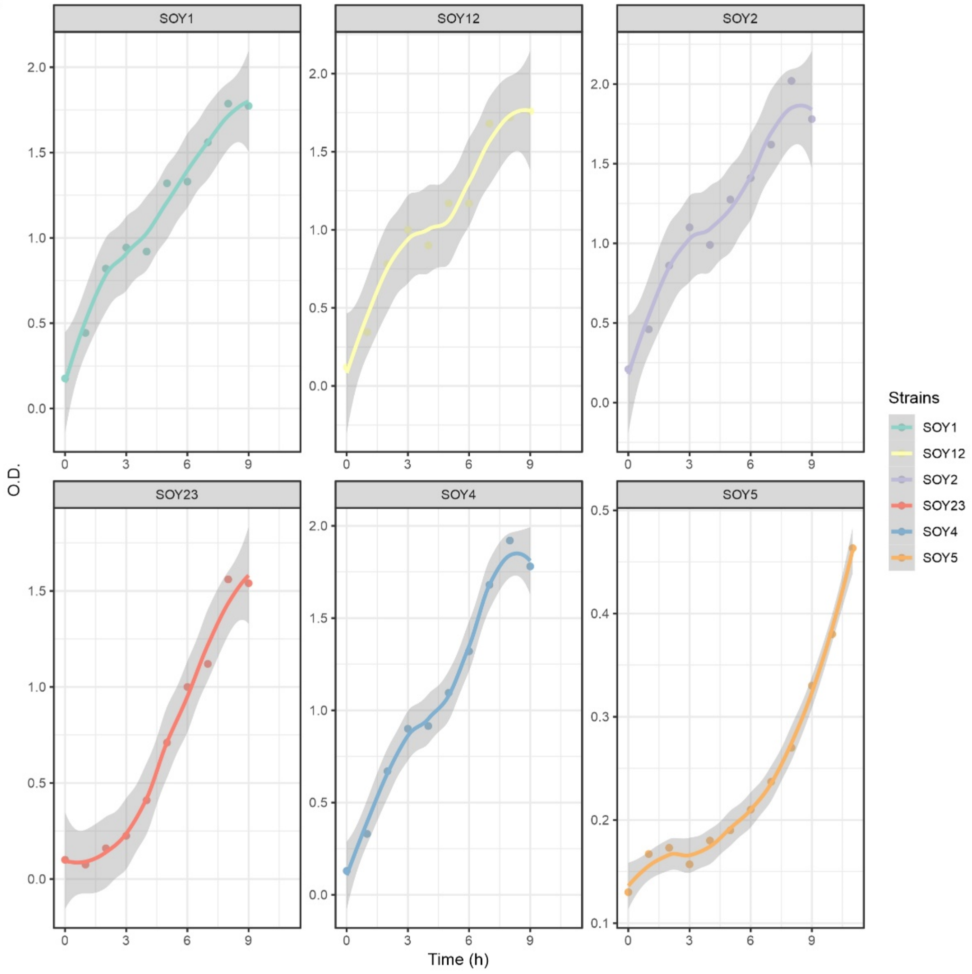

B

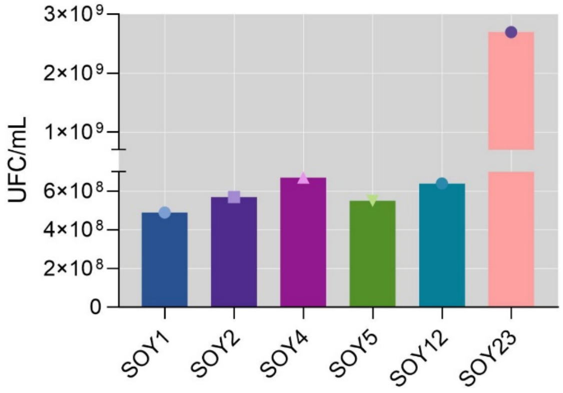

Figure S3. Standard growth curve for isolates. **A**). Dispersion curve. The x-axis shows time in hours, and the y-axis shows absorbance in nm. **B**). Bar graph depicting plaque growth after 3 hours. The X-axis represents isolates, the y-axis represents colony-forming units (CFU) per ml.

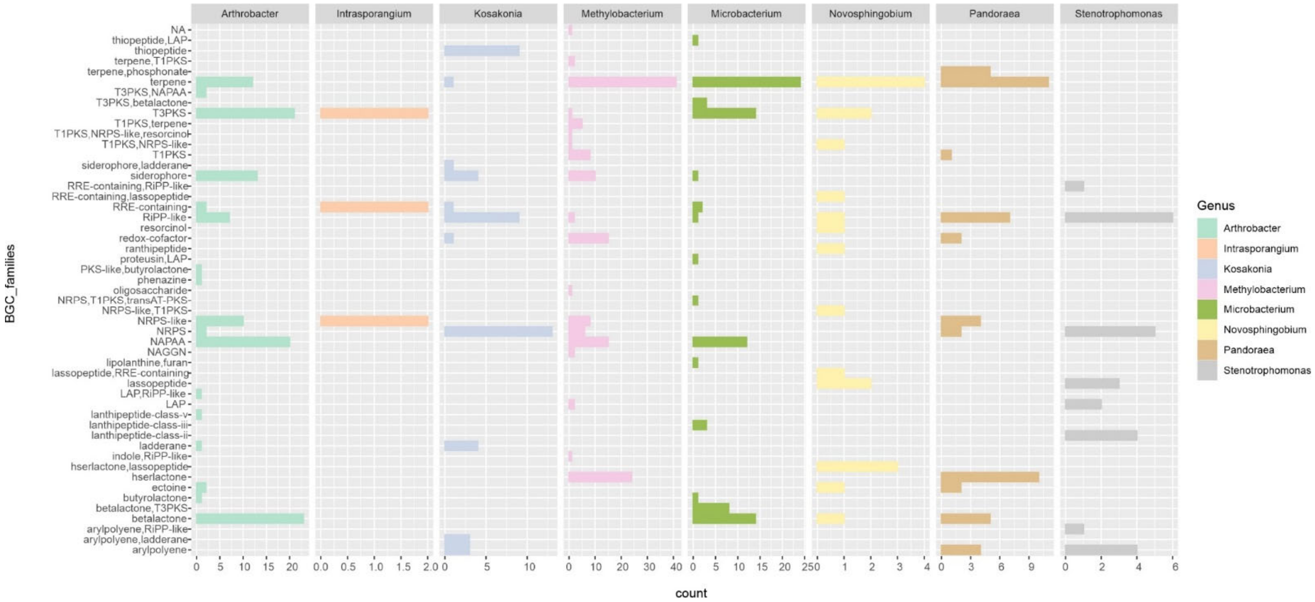

Figure S4. Biosynthetic gene clusters identified from the 169 publicly genomic sequences available for soil bacteria.

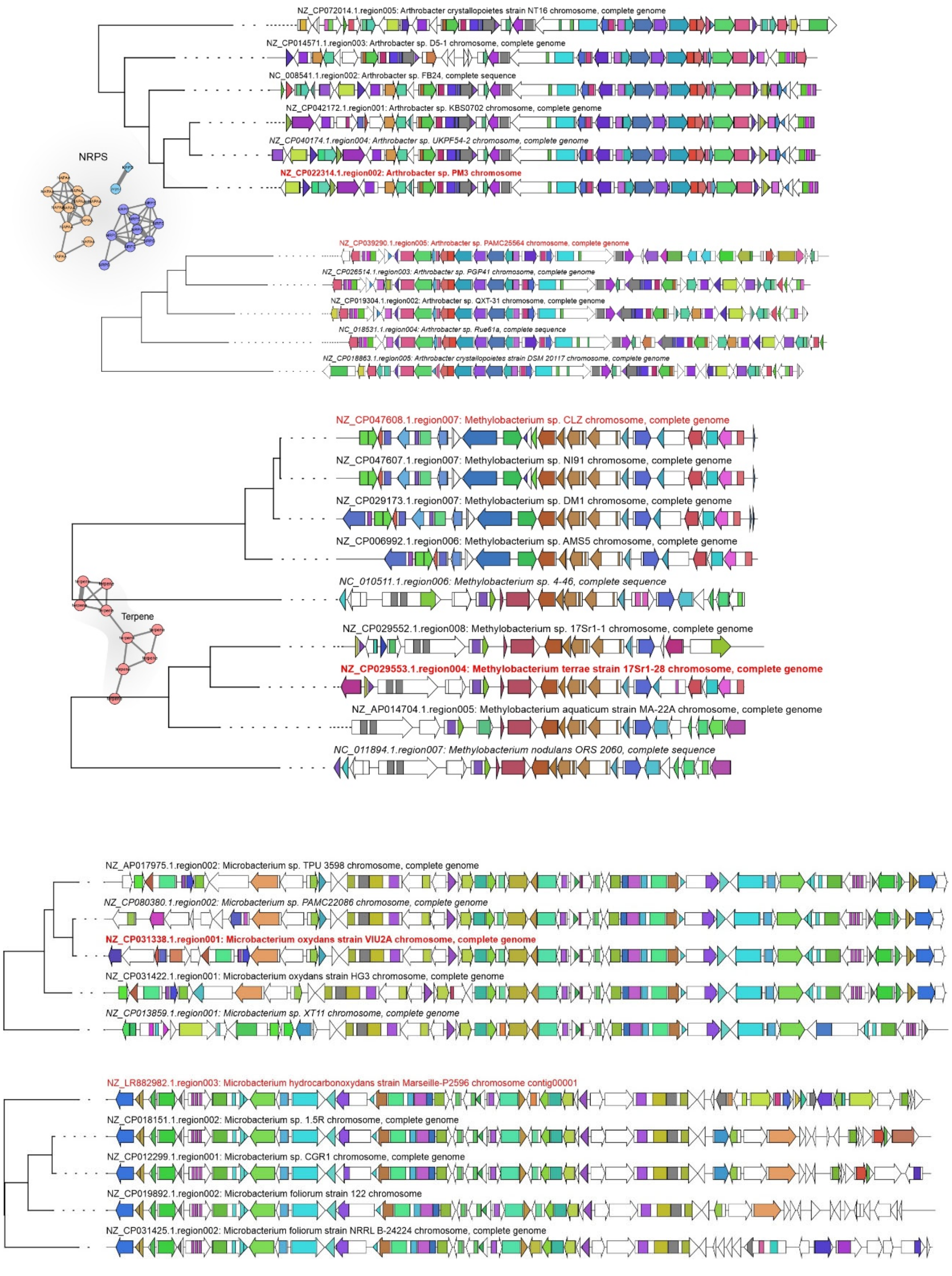

Figure S5. CORASON phylogeny of Biosynthetic gene cluster identified from the 169 publicly genomic sequences available for soil bacteria.
